# Co-exposure to lead, mercury, and cadmium induces neurobehavioral impairments in mice by interfering with dopaminergic and serotonergic neurotransmission in the striatum

**DOI:** 10.3389/fpubh.2023.1265864

**Published:** 2023-11-07

**Authors:** Sarita Pyatha, Haesoo Kim, Daeun Lee, Kisok Kim

**Affiliations:** College of Pharmacy, Keimyung University, Daegu, Republic of Korea,

**Keywords:** metal coexposure, neurobehavioral toxicity, dopamine, serotonin, striatum

## Abstract

Humans are exposed to lead (Pb), mercury (Hg), and cadmium (Cd) through various routes, including drinking water, and such exposure can lead to a range of toxicological effects. However, few studies have investigated the toxic effects of exposure to mixtures of metals, particularly in relation to neurotoxicity. In this study, 7-week-old male mice were exposed to Pb, Hg, and Cd individually or in combination through their drinking water for 28 days. The mice exposed to the metal mixture exhibited significantly reduced motor coordination and impaired learning and memory abilities compared to the control group and each of the single metal exposure groups, indicating a higher level of neurotoxicity of the metal mixture. The dopamine content in the striatum was significantly lower in the metal mixture exposure group than in the single metal exposure groups and the control group. Furthermore, compared to the control group, the metal mixture exposure group showed a significantly lower expression level of tyrosine hydroxylase (TH) and significantly higher expression levels of dopamine transporter (DAT), tryptophan hydroxylase 1 (TPH1), and serotonin reuptake transporter (SERT). Notably, there were no significant differences in SERT expression between the single metal exposure groups and the control group, but SERT expression was significantly higher in the metal mixture exposure group than in the single metal and control groups. These findings suggest that the key proteins involved in the synthesis and reuptake of dopamine (TH and DAT, respectively), as well as in the synthesis and reuptake of serotonin (TPH1 and SERT, respectively), play crucial roles in the neurotoxic effects associated with exposure to metal mixtures. In conclusion, this study demonstrates that simultaneous exposure to different metals can impact key enzymes involved in dopaminergic and serotonergic neurotransmission processes, leading to disruptions in dopamine and serotonin homeostasis and consequently a range of detrimental neurobehavioral effects.

## Introduction

Heavy metals are inorganic environmental pollutants that tend to persist in the environment for prolonged periods. Diverse human activities and natural processes contribute to widespread metal contamination, leading to accumulation in the environment and affecting all aspects of the biosystem, including human health (1). Despite the well-known detrimental effects of heavy metals, their utilization and accumulation in the environment continue to increase (2). Lead (Pb), mercury (Hg), and cadmium (Cd) are commonly used heavy metals that can have considerable biological implications. These metals are of particular concern because of their toxicity and persistence in the environment, and they have been listed among the 10 most harmful environmental toxicants by the Agency for Toxic Substances and Disease Registry (3). Furthermore, the World Health Organization (WHO) has identified these three metals as major public health issues and has set drinking water guidelines of 10 μg/L, 6 μg/L, and 3 μg/L, respectively (4).

Previous studies have shown that Pb is a neurotoxicant that can cause impaired motor function, behavioral dysfunction, and cognitive decline in humans and rats (5–7). Chronic exposure to Cd is associated with various adverse effects including memory loss, motor neuron disease, behavioral changes, delayed psychomotor activity in workers (8–10), and impaired memory and learning in experimental animals (11). Hg is a well-known neurotoxicant that can disrupt normal central nervous system development (12). Even low-level exposure to Hg can have a negative impact on motor function (13), and animals treated with Hg reportedly exhibit behavioral deficits and memory impairment (14).

Several studies have revealed the harmful effects of individual metals. However, metals rarely occur alone in nature; rather, they occur as mixtures (15). Metals can enter the human body through various routes, including drinking water, food, and air (16). Pb, Hg, and Cd have been simultaneously detected in the serum, urine, and blood of populations living in different parts of the world (17, 18). Several studies have evaluated the toxic effects of coexposure to metals on the renal (19), respiratory (20, 21), and cardiovascular (22) systems as well as on pediatric health (23, 24); however, few such studies have focused on the nervous system (25, 26). Pb, Hg, and Cd are typical neurotoxic contaminants that have various neurological adverse effects upon exposure alone, but few studies have investigated the neurotoxic effects of concurrent exposure to Pb, Cd, and Hg.

Although the precise mechanisms underlying the neurotoxicity induced by Pb, Hg, and Cd are not fully understood, studies have shown that these metals can penetrate the brain by various routes and have neurotoxic effects by modulating multiple neurotransmitter systems (27–29). Metals can accumulate in and affect various parts of the brain, but one of the main areas is the striatum (30–32), a major component of the basal ganglia, which is responsible for coordination of voluntary movement. It also plays a role in learning and memory processes by receiving cognitive and sensory inputs (33). Hence, striatal damage is a common feature in several neurodegenerative diseases, encompassing Huntington’s disease, Parkinson’s disease, and the Tauopathies (34). Monoamine neurotransmitters, including dopamine and serotonin, play crucial roles in regulating brain functions, including motor control, memory, and learning (35). Therefore, it is important to understand the extent of neurotoxicity resulting from concurrent exposure to different metals by assessing striatum-dependent functions. In addition, it is important to investigate whether these functional effects are caused by changes in the dopaminergic and/or serotonergic systems.

To investigate the neurotoxicological implications of coexposure to multiple metals in the present study, we compared the effects of individual exposure to Pb, Hg, or Cd with the effects of exposure to a mixture of these metals (Pb + Hg + Cd). C57BL/6 mice were administered the metals individually or in combination through their drinking water because this route reflects a common mode of metal exposure in humans (36). The administered doses were determined based on previous studies and WHO guidelines for drinking water (37). Behavioral tests were performed to assess neurobehavioral dysfunction, and changes in the neurotransmitter systems in the striatum were analyzed to elucidate the underlying mechanisms of action.

## Materials and methods

### Animals and treatment

**Figure 1:**
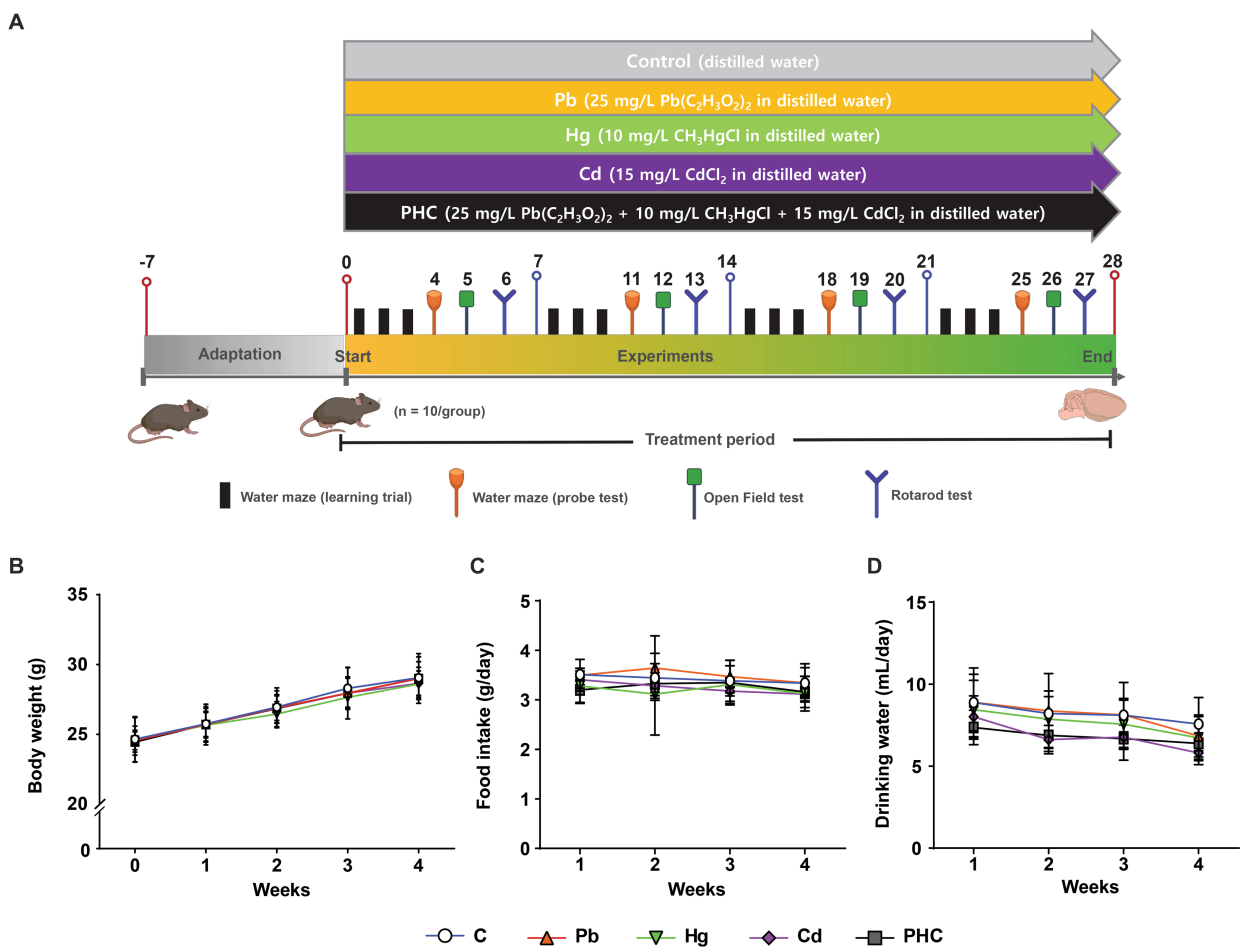
Experimental design and physiological parameters. **(A)** Schematic representation of the experimental protocol depicting the exposure of mice to individual and combined treatments of Pb, Hg, and Cd for a duration of 28 days. Behavioral tests were conducted during the treatment period. **(B)** Body weight. **(C)** Total food intake. **(D)** Total consumption of drinking water. Values are expressed as mean ± SD (*n* = 10). C, control; Pb, lead; Hg, mercury; Cd, cadmium; PHC, Pb + Hg + Cd.

Seven-week-old C57BL/6 male mice (17.73 ± 0.06 g) were obtained from Samtako Bio Korea (Osan, Korea). The study protocol was approved by the Institutional Animal Care and Use Committee of Keimyung University (approval no. KM2022–002). All animal procedures were conducted in accordance with the accepted National Institutes of Health recommendations for the care and use of laboratory animals. The animals were housed (two mice of the same treatment group per cage) under a 12 h light–dark cycle, temperature of 20°C to 22°C, and relative humidity of 55% ± 5% with sufficient water and food. After an acclimation period of 1 week, the animals were randomly divided into five groups (n = 10 each): Pb, Hg, Cd, PHC (Pb + Hg + Cd), and control. The mice in the Pb, Hg, Cd, and PHC groups were exposed to lead acetate (25 mg/L Pb(C_2_H_3_O_2_)_2_ in distilled water), methylmercury chloride (10 mg/L CH_3_HgCl in distilled water), cadmium chloride (15 mg/L CdCl_2_ in distilled water), and a mixture of all three, respectively, through their drinking water (Figure 1A) for a subacute period of 28 days. The concentration of metal used in this study was based on previous studies (38–40) that evaluated neurotoxic effects. The lead acetate, methylmercury chloride, and cadmium chloride used in the experiments were purchased from Sigma-Aldrich (St. Louis, MO, United States). The metal solution was freshly prepared in distilled water before each use, ensuring the solvability and stability of the treatment solution. Control group animals were provided only distilled deionized water. Total food and water intake and body weight were measured twice a week. In addition, the animals were subjected to behavioral experiments during the treatment period. Then they were euthanized with carbon dioxide on day 29, and the striatum was dissected from the brain and stored at −80°C for future use.

### Rotarod test

A rotarod treadmill (Jeungdo Bio & Plant, Seoul, Korea) was used to evaluate motor coordination. The animals were trained on the apparatus for 3 days prior to the trial. The rotarod was programmed to gradually increase the acceleration from 10 to 30 rotations/min over a 10 s period, and the total time for the test was set to 300 s. During the trial, the animals were positioned on the horizontal rod, and the falling-off time of each animal was recorded. Each trial was performed three times per day once a week. The mean time of the three trials was used for analyzes.

### Open field test

An open field test was performed to assess the effect of metal exposure on the animals’ locomotor and behavioral activity, which can be correlated with locomotive function. The experiment was conducted on the same day every week during the treatment period. The apparatus used consisted of a 50 × 50 cm square area with a 50 cm high black opaque wall. The test was started 5 min after the mouse was placed in the square area to allow for adaptation. Then the mouse was allowed to explore for 15 min, and simultaneous measurement of movement, navigation, and anxiety was captured with a camera and SMART video tracking software (Panlab, Barcelona, Spain). The following parameters were measured during the experiment: total distance traveled (locomotor and exploratory activity), total time spent within and number of entries into the center (inversely correlated with anxiety level), and time spent in the corners and at the sides. Then the mean value of each parameter was statistically analyzed. After each test, all fecal pellets were manually removed and the arena was wiped with 70% alcohol to eliminate odor hints.

### Morris water maze test

The Morris water maze was divided into two parts: a learning trial and a probe test. The learning trial was conducted twice a day for 3 consecutive days, and the probe test was conducted after each learning trial. Each learning trial + probe test was conducted once a week to assess the progressive effect of metal exposure on spatial learning and memory ability. A white circular pool (120 cm diameter, 30 cm height) was filled with tap water (26–27°C, with white food coloring added for invisibility), and a 5 cm diameter platform was placed in the southwest quadrant (0.5 cm below the water level, 25 cm away from pool wall). For each learning trial, the animal was released into the water facing the pool wall from the northeastern quadrant and given a maximum of 90 s to find the escape platform. At the end of each trial, the animal was allowed to either stay on the platform or be guided to the platform (if the animal failed to locate the platform within 90 s) for 10 s. Between and after each learning trial, the animal was removed from the platform and placed on a towel to dry. The latency of locating the escape platform was recorded during the trial; if the platform could not be found, the full 90 s latency score was allocated. The platform was removed on the probe test day. The animal was released from the same quadrant as before and given 120 s for the probe test to assess its capacity for memory retention. The SMART video tracking system recorded the following parameters during the test: latency from first entrance to target zone (quadrant with escape platform during trial test), time spent in platform area, and number of platform area crossings (suggesting the level of memory retention following the learning period).

### Measurement of dopamine concentration

The dopamine concentration in striatum tissue homogenates was measured using a mouse dopamine enzyme-linked immunosorbent assay kit (Cusabio, Houston, TX, United States) according to the manufacturer’s instructions. Striatal tissue (100 mg) was washed with 1X PBS, homogenized in 1 mL of 1X PBS, and stored at −20°C overnight. After two freeze–thaw cycles, the homogenates were centrifuged at 5000 x g for 5 min at 4°C. The supernate was removed and immediately assayed. A microplate reader (Infinite 200 PRO; Tecan, Männedorf, Switzerland) was used to measure absorbance at a wavelength of 450 nm. The assay had a detection range of 5 to 1,000 pg./mL and a sensitivity of 2.5 pg./mL.

### Real-time polymerase chain reaction array

Total RNA was extracted from the striatum of the animals for quantification of mRNA using the NucleoSpin RNA kit (Macherey-Nagel, Düren, Germany) following the manufacturer’s protocol. A NanoDrop 2000 spectrophotometer (Thermo Scientific, Waltham, MA, United States) was used for RNA sample quantification, and an iScript cDNA Synthesis kit (Bio-Rad, Hercules, CA, United States) was used for reverse-transcription of total RNA from each sample; each device was used according to the manufacturer’s protocol. The cDNAs were characterized using a mouse dopamine and serotonin pathway polymerase chain reaction (PCR) array (RT^2^ Profiler PCR Array, Cat. No. PAMM-158Z; Qiagen, Hilden, Germany) and RT^2^ SYBR Green ROX FAST Mastermix (Qiagen) on a CFX96 real-time PCR system (Bio-Rad) according to the instructions for the RT^2^ Profiler PCR Array. Data were analyzed using the Ct method, and *β*-actin was used as a housekeeping gene for normalization. For each gene, the fold change was calculated as the difference in gene expression between the control and PHC groups. The cut-off values of the fold change for significant upregulation and downregulation were set to >1.75 and < −1.75, respectively.

### Quantitative real-time PCR

Quantitative real-time PCR was used to validate significantly upregulated or downregulated genes (fold change >1.75 or < −1.75, respectively) in the metal exposure groups and the control group. cDNA was characterized using the SsoAdvanced Universal SYBR Green Supermix kit (Bio-Rad) on a CFX96 real-time PCR system (Bio-Rad). Then the following genes were analyzed using β-actin (NM_007393.5) as the reference gene: tyrosine hydroxylase (*TH*, NM_009377), dopamine transporter (*SLC6A3*, *DAT*, NM_010020), dopamine receptor D4 (*DRD4*, NM_007878), tryptophan hydroxylase 1 (*TPH1*, NM_009414), and serotonin transporter (*SLC6A4*, *SERT*, NM010484) (Supplementary Table S1). *β*-actin was used as a housekeeping gene for normalization. CFX Manager Software (Bio-Rad) was used to analyze the quantification cycle data obtained from the PCR amplification and thus acquire the expression levels of the amplified genes.

### Western blotting analysis

The striatum of the animals was homogenized using radioimmunoprecipitation assay buffer (Sigma-Aldrich), 1% protease inhibitor cocktail, and phosphatase inhibitor cocktail. The supernatant was collected from the homogenate after centrifugation for 20 min at 4°C. Then the Bradford method was used to estimate the protein concentration with bovine serum albumin as the standard. A 10 μg aliquot of the protein sample was separated by 10% sodium dodecyl sulfate-polyacrylamide gel electrophoresis and transferred onto a nitrocellulose membrane. Immunoblotting was performed using the following primary antibodies: mouse anti-TH monoclonal antibody (1:150,000 dilution; Chemicon International, Temecula, CA, United States), rat anti-DAT monoclonal antibody (1:1000 dilution; Santa Cruz Biotechnology, Dallas, TX, United States), mouse anti-DRD4 monoclonal antibody (1:1000 dilution; Santa Cruz Biotechnology), rabbit anti-SERT polyclonal antibody (1:1000 dilution; Abcam, Cambridge, United Kingdom), and rabbit anti-TPH1 monoclonal antibody (1:500 dilution; Abcam) with *β*-actin as a loading control. Following incubation with horseradish peroxidase-conjugated rabbit or mouse secondary antibodies (Santa Cruz Biotechnology), the immunoreactive bands were visualized using enhanced chemiluminescence Western blotting detection reagents (Amersham Biosciences, Piscataway, NJ, United States) and the LAS 4000 system (GE Healthcare, Chicago, IL, United States) X-ray film. The band intensity was quantified using ImageJ software (National Institutes of Health, Bethesda, MD, USA), with β-actin used as an internal control for immunoblotting.

### Statistical analysis

All data are presented as mean ± standard error of the mean (SEM) or mean ± standard deviation (SD). Statistical differences among groups were assessed using one-way analysis of variance followed by Duncan’s *post hoc* test. Statistical analyzes were conducted using SAS v.9.4 statistical software (SAS Institute Inc., Cary, NC, United States), and *p* < 0.05, *p* < 0.01, or *p* < 0.001 was considered statistically significant.

## Results

### General observation, body weight, and total food and drinking water intake

No physical evidence of toxicity was observed in any animals during the study period, indicating that the animals tolerated both individual and combined heavy metals in their drinking water. This was supported by the gradual increase in body weight of all animals throughout the study (Figure 1B). The body weights of mice in the Pb, Hg, Cd, or PHC group were not significantly different from those of the mice in the control group throughout the experimental period. In addition, the total consumption of food and drinking water was similar across all groups, with no significant differences between the control and treatment groups (Figures 1C,D). Based on the water consumption data, the mean daily metal intake in the Pb, Hg, Cd, and PHC groups was determined to be 7.52 mg/kg bw/day, 2.88 mg/kg bw/day, 3.81 mg/kg bw/day, and 12.72 mg/kg bw/day, respectively.

### Motor coordination

**Figure 2:**
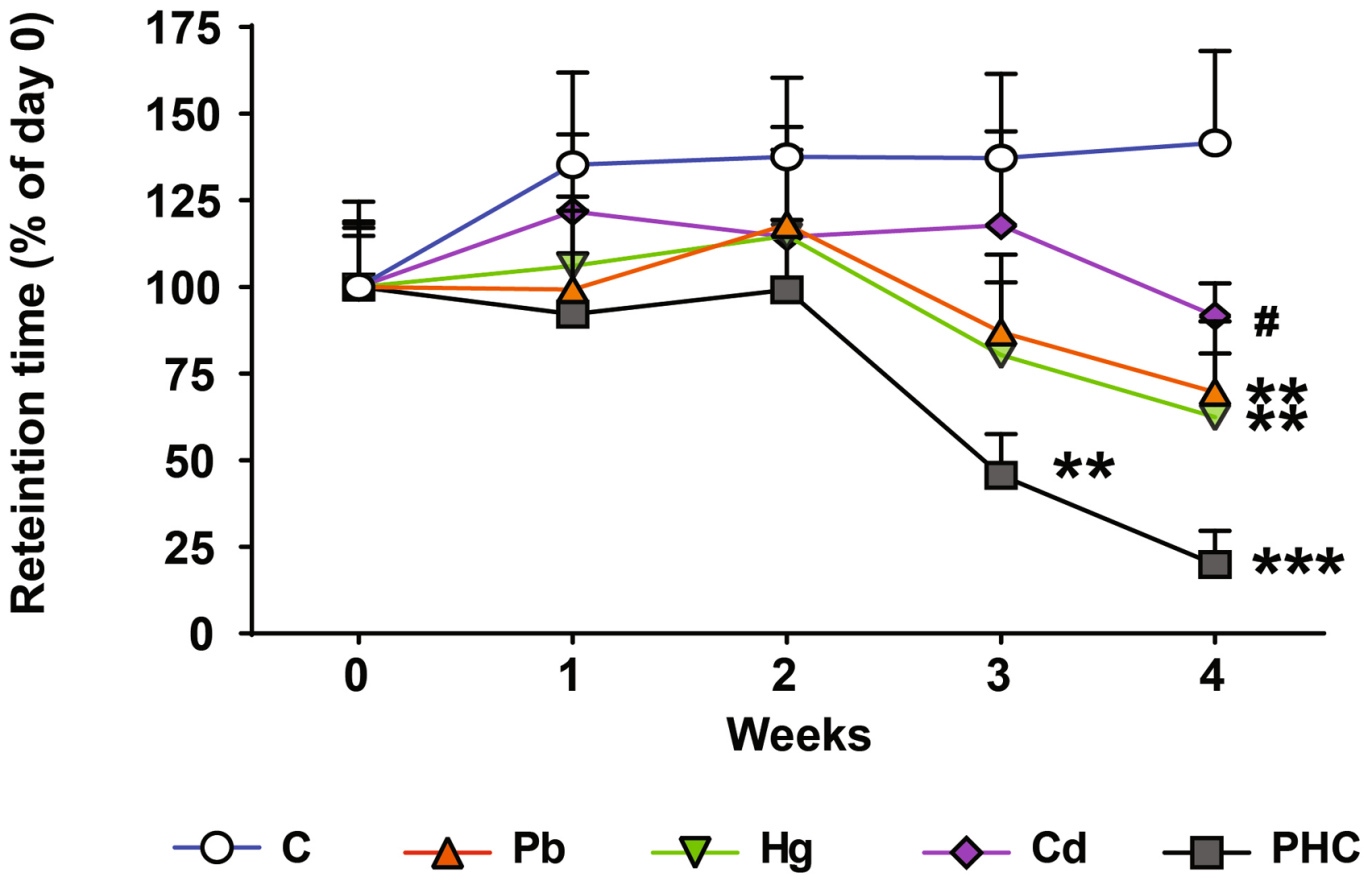
Effects of metal exposure on motor coordination. Retention time was measured using the rotarod test to assess motor coordination. The error bar reflects the SEM for each group (*n* = 10); ^**^p < 0.01, ^***^p < 0.001 compared to control group; ^#^*p* < 0.05 compared to metal mixture (PHC) group. C, control; Pb, lead; Hg, mercury; Cd, cadmium; PHC, Pb + Hg + Cd.

The animals’ motor coordination ability was assessed before and during the administration of drinking water containing Pb, Hg, and Cd using the rotarod test. The mice in the Pb and Hg groups showed a significant decrease in the retention time at week 4; the retention time percentage decreased to 69.58 and 62.41% in the Pb and Hg groups, respectively, compared to the control group [F (4, 45) = 6.03, *p* < 0.01] (Figure 2). Motor coordination in the PHC group decreased significantly from week 3, with retention time percentages of 45.56% (*p* < 0.01) at week 3 and 19.91% (*p* < 0.001) at week 4 compared to the control group. The motor coordination in the combined exposure group (PHC group) showed a greater decrease than that in the single metal exposure groups, particularly the Pb exposure group (*p* < 0.05).

### Locomotor activity

**Figure 3:**
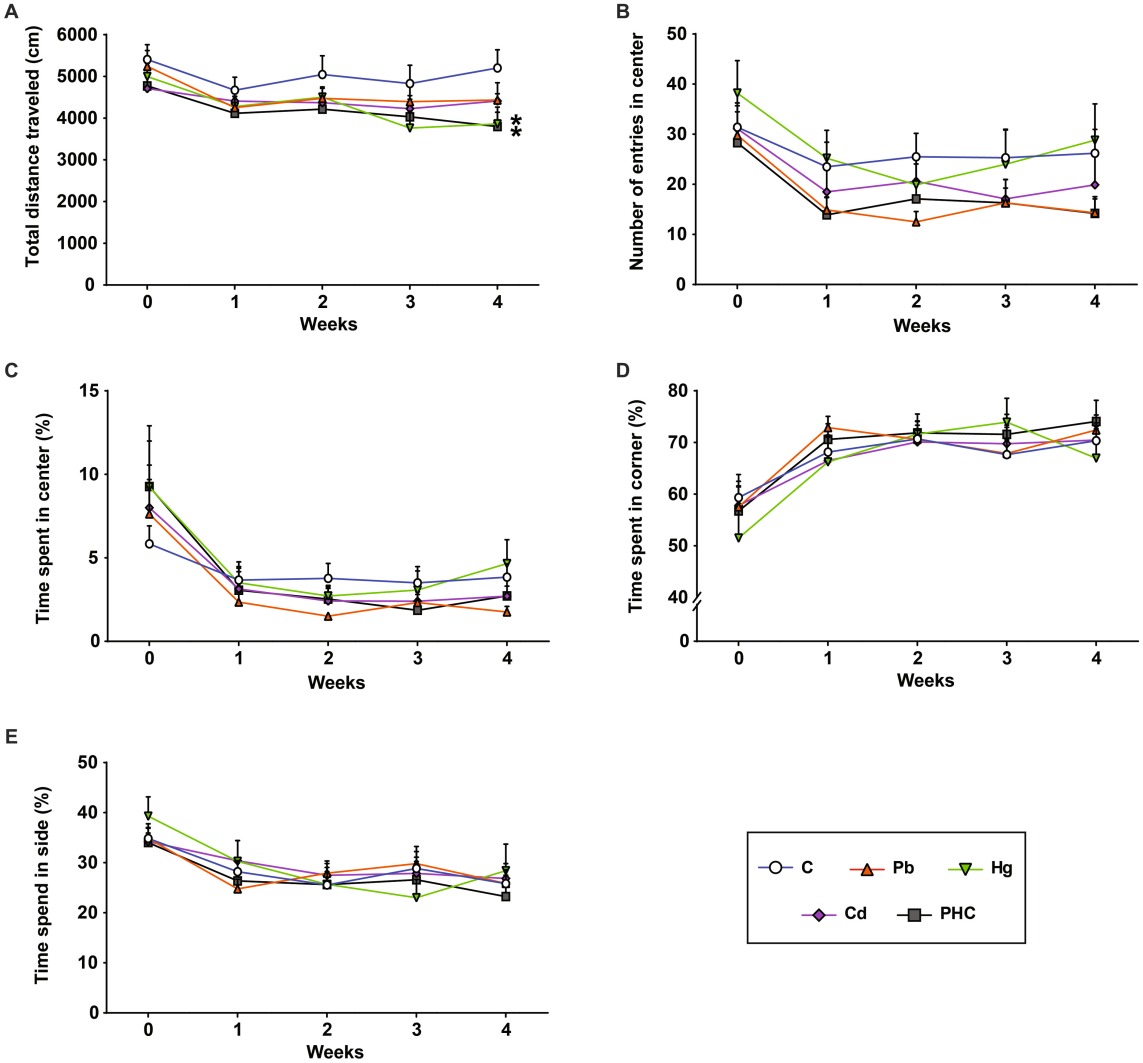
Effects of metal exposure on locomotor activity. **(A)** Total distance traveled, **(B)** number of entries into the center, **(C)** time spent at the center, **(D)** time spend in the corners, and **(E)** time spent at the sides were measured in the open field test to assess locomotor and exploratory activity in mice. The error bar reflects the SEM for each group (*n* = 10); ^*^p < 0.05 compared to control group. C, control; Pb, lead; Hg, mercury; Cd, cadmium; PHC, Pb + Hg + Cd.

The effects of exposure to Pb, Hg, and Cd individually and as a mixture on the locomotor activity of mice was assessed using an open field test. The total distance traveled in the Hg group and PHC group gradually decreased throughout the administration period and was significantly less at week 4 than that in the control group (*p* < 0.05) (Figure 3A). However, there were no significant differences in the number of entrances to the central area or the total time spent in the center, corners, or sides between the control and treatment groups (Figures 3B–E).

### Learning and memory function

**Figure 4:**
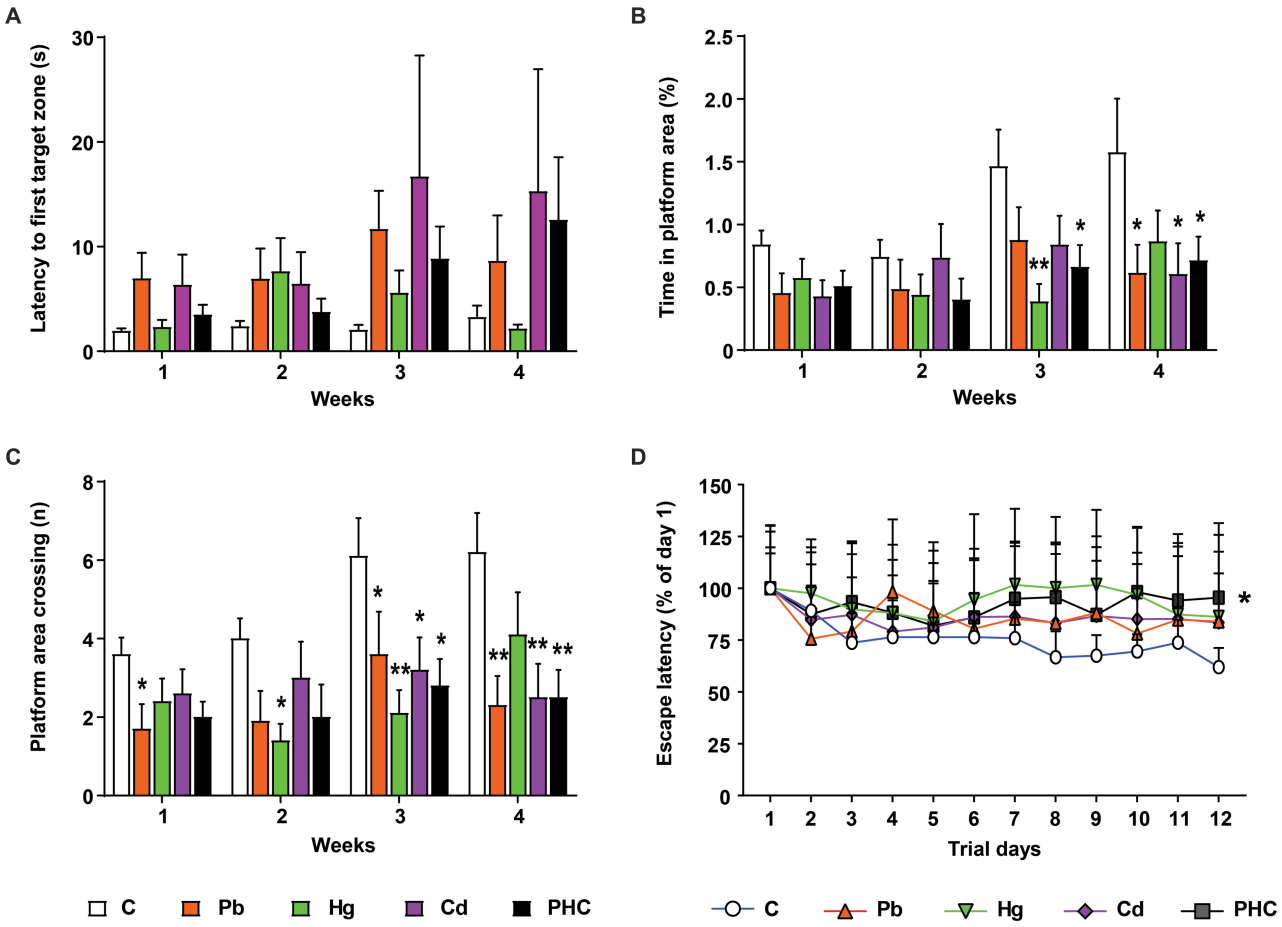
Effects of metal exposure on learning and memory ability in mice. Using the Morris water maze test, the **(A)** latency to first entrance to the target zone, **(B)** time spent in the platform area, **(C)** number of platform area crossings, and **(D)** escape latency time were analyzed to assess learning and memory ability in mice. The error bar reflects the SEM for each group (*n* = 10); ^*^p < 0.05, ^**^p < 0.01 compared to control group. C, control; Pb, lead; Hg, mercury; Cd, cadmium; PHC, Pb + Hg + Cd.

The Morris water maze test was conducted to evaluate the learning and memory function of mice after exposure to metals. In the probe test, there were no significant differences in latency of the first entrance to the target zone or platform zone between the metal-treated groups and the control group (Figure 4A). However, the time spent in the platform area and the number of platform area crossings showed significant or marginally significant decreases in the metal-treated groups from the third week of administration compared to the control group (Figures 4B,C). By contrast, there were no significant differences in the probe test between the single metal exposure groups and the PHC group. In the learning trial, the escape latency time was significantly longer in the PHC group than in the control group (*p* < 0.05), indicating that the mice in the PHC group took a longer time to find the platform (Figure 4D).

### Dopamine concentration

**Figure 5:**
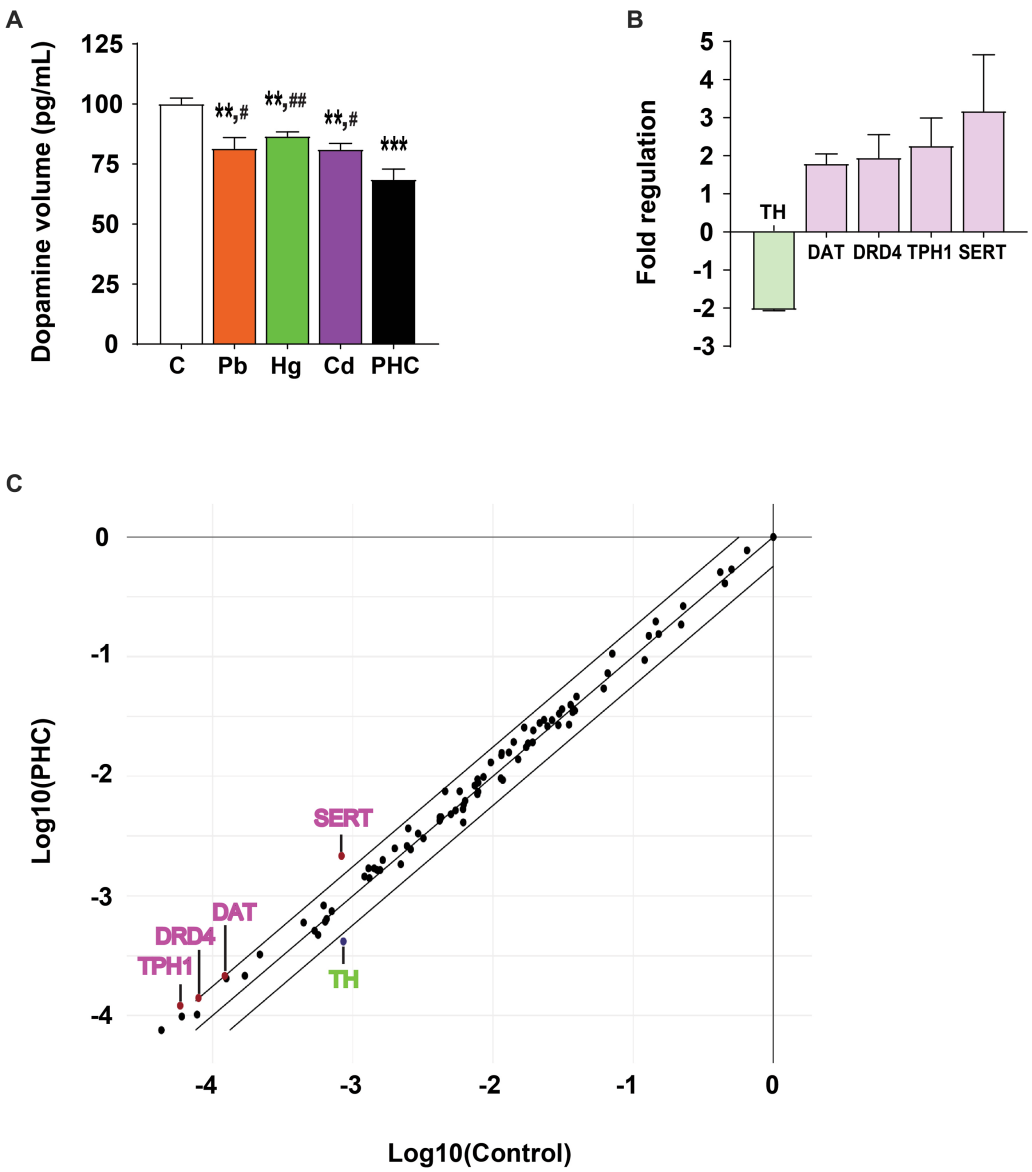
Effect of metal exposure on dopamine content and gene expression in PCR array. **(A)** Relative dopamine content in the striatum of mice. The error bar represents the mean ± SEM (*n* = 5). **(B)** Fold regulation and **(C)** scatter plot of genes exceeding the cut-off value (>1.75 or < −1.75) among 84 genes involved in dopaminergic and serotonergic neurotransmission processes in the striatum of the metal mixture (PHC) group. Gene expression was normalized against *β*-actin mRNA expression, and the error bar represents the mean ± SEM (*n* = 3); ^**^p < 0.01, ^***^p < 0.001 compared to control group; ^#^*p* < 0.05, ^##^*p* < 0.01 compared to PHC group. C, control; Pb, lead; Hg, mercury; Cd, cadmium; PHC, Pb + Hg + Cd.

Exposure to a single metal or a mixture of metals significantly decreased the dopamine concentration in the striatum of mice, indicating damage to the dopaminergic neurotransmitter system (Figure 5A). The dopamine concentrations in the Pb, Hg, and Cd, groups were significantly reduced to 81.5, 86.5, and 81.1% of that in the control group, respectively [F (4, 20) = 11.80, *p* < 0.01]. The striatal dopamine content in the PHC group was even lower (68.5% of that in the control group) (*p* < 0.001). In addition, the striatal dopamine content in the PHC group was significantly lower than that in all single metal exposure groups (*p* < 0.01 or *p* < 0.05).

### PCR array

To explore the molecular basis of PHC toxicity, mRNA expression patterns of the 84 key genes related to the dopaminergic and serotonergic pathways were compared between the control and PHC groups using real-time PCR array. Gene expressions of markers in the striatum were compared between the PHC group and the control group using fold-change analysis (Supplementary Table S2). The genes with a fold regulation value greater than the cut-off value of 1.75 were *DAT*, *DRD4*, *TPH1*, and *SERT*, and the gene with a fold regulation value of less than −1.75 was *TH* (Figures 5B,C). In particular, compared to the control, exposure to the metal mixture significantly downregulated *TH* expression by 0.49-fold (*p* < 0.05) and significantly upregulated *SERT* expression by 2.60-fold (*p* < 0.02).

### Gene expression in striatum

**Figure 6:**
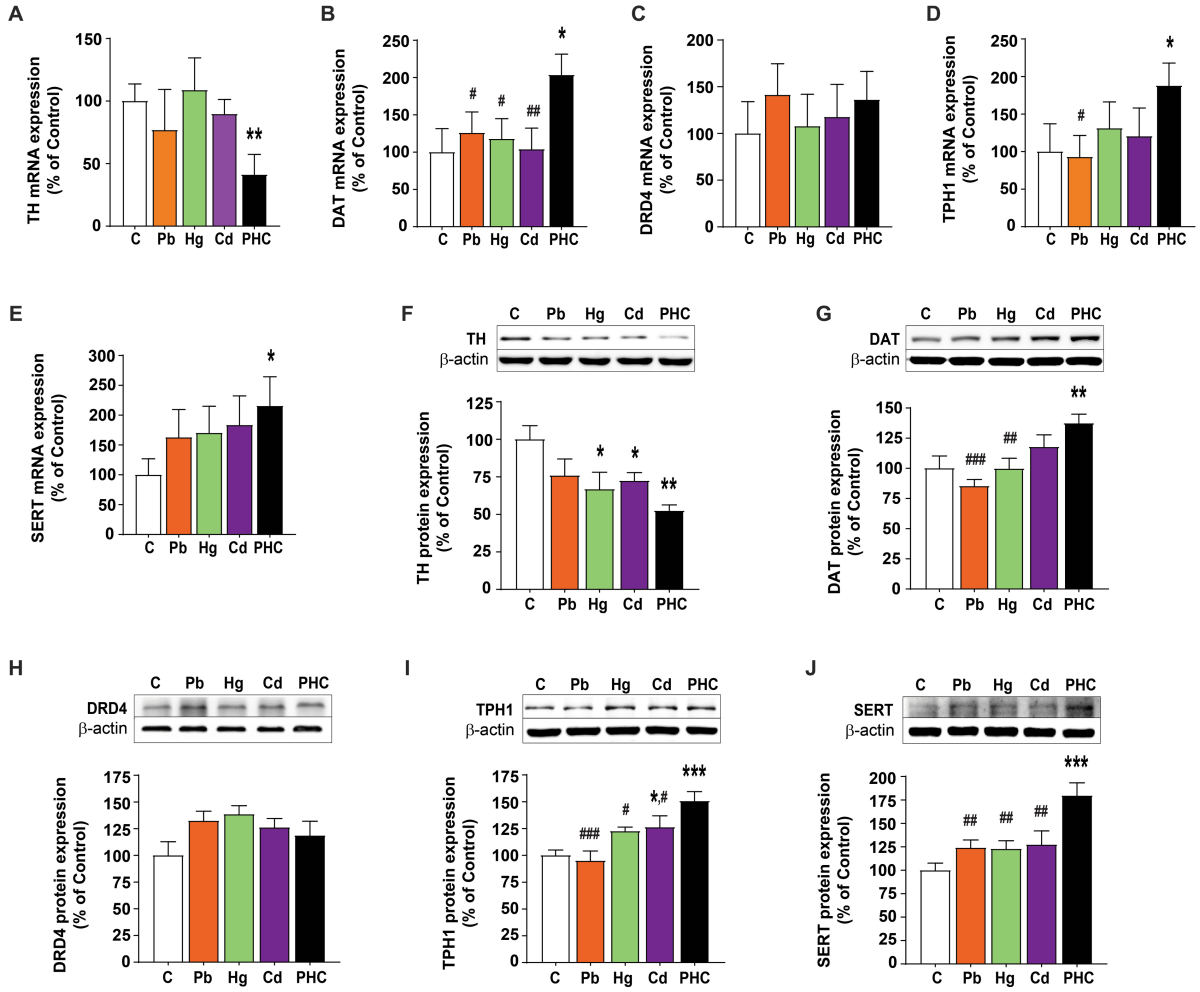
Gene and protein expressions in the striatum of metal-exposed mice. Relative gene expression levels of **(A)** TH, **(B)** DAT, **(C)** DRD4, **(D)** TPH1, and **(E)** SERT in the striatum of mice. Relative protein expression levels of **(F)** TH, **(G)** DAT, **(H)** DRD4, **(I)** TPH1, and **(J)** SERT in the striatum of mice. Each panel shows a representative Western blot band (top) and densitometric analysis of the protein bands in the control and metal-treated groups (bottom). The error bar represents the SEM for each group (*n* = 5); ^*^p < 0.05, ^**^p < 0.001 compared to control group; ^#^*p* < 0.05, ^##^*p* < 0.01, ^###^*p* < 0.001 compared to metal mixture (PHC) group. TH, tyrosine hydroxylase; DAT, dopamine transporter; DRD4, dopamine receptor D4; TPH1, tryptophan hydroxylase 1; SERT, serotonin transporter; C, control; Pb, lead; Hg, mercury; Cd, cadmium; PHC, Pb + Hg + Cd.

Based on the data obtained from the PCR array analysis, real-time quantitative PCR analysis was performed to confirm the mRNA expression patterns of factors related to the dopaminergic and serotonergic systems following metal exposure. Similar to the PCR array results, *TH* mRNA expression was significantly lower in the PHC than control group (*p* < 0.01), but there were no significant differences in *TH* mRNA expression between the single metal exposure groups and the control group (Figure 6A). *DAT* mRNA expression was not significantly different between the single metal exposure groups and the control group. However, *DAT* mRNA expression was significantly higher in the PHC group than in the single metal exposure groups and the control group (*p* < 0.05) (Figure 6B). By contrast, *DRD4* mRNA expression was not significantly different between the single and mixed metal exposure groups (Figure 6C). Similar to the results of the PCR array, *TPH1* mRNA expression was significantly higher in the PHC group than in both the Pb exposure group and the control group (*p* < 0.05), and mRNA expression of *SERT* was significantly higher in the PHC than control group (*p* < 0.05) (Figures 6D,E).

### Protein expression in striatum

To determine the effects of Pb, Hg, Cd, and the metal mixture on the expression of dopaminergic and serotonergic systems at the protein level, Western blotting analysis of the striatum was performed in the control and metal-treated groups. Consistent with the mRNA expression results, exposure to the metal mixture significantly reduced the TH protein expression level in the striatum by 47.6% compared to the control group [F (4, 20) = 3.98, *p* < 0.05]. Exposure to Pb or Hg also significantly reduced the TH protein expression level compared to the control, but the reduction was more pronounced in the PHC group (Figure 6F). The expression of DAT protein was significantly higher in the PHC group than in the Pb or Hg group and control group (*p* < 0.01 or *p* < 0.001) (Figure 6G). However, consistent with the quantitative PCR results, metal treatment did not have a significant effect on the protein expression of DRD4 (Figure 6H). Furthermore, the TPH1 protein expression pattern was similar to the TPH1 mRNA expression pattern, with significant increases in the PHC and Cd groups compared to the control group. In addition, TPH1 protein expression in the PHC group was significantly higher than that in the single metal exposure groups and the control group (*p* < 0.05 or *p* < 0.001) (Figure 6I). Similarly, there were no significant differences in SERT mRNA expression between the single metal exposure groups and the control group; however, SERT protein expression was significantly higher in the PHC group than in the control group and single metal exposure groups (*p* < 0.01 or *p* < 0.001) (Figure 6J).

## Discussion

This study investigated the neurotoxic effects of combined exposure to harmful metals (Pb, Hg, and Cd) on neurobehavioral function through changes in neurotransmitter systems. Overall, the results showed that, compared to exposure to single metals, exposure to the combination of Pb, Hg, and Cd has more severe neurotoxic effects as evidenced by altered neurotransmitter stability and neurobehavioral dysfunction. Body weight is an indicator of an individual’s overall health condition (41). In this study, the body weight of the animals in all groups gradually increased throughout the study period. In addition, there were no significant differences in the total intake of food or drinking water between the control and treatment groups. Therefore, these results allow us to exclude undernutrition as a major confounding factor for behavioral changes (42).

A battery of neurofunctional tests was conducted to assess motor coordination, locomotor activity, and learning and memory function. Neurological impairment, indicated by loss of equilibrium (falling), was more pronounced in the mixture group than in the single metal groups. Consistent with previous studies (43–45), the retention time of the mice in the Pb or Hg group significantly decreased at week 4. By contrast, the mice in the PHC group showed evidence of motor coordination and motor skill learning impairment from 3 weeks of treatment, and these abnormalities had significantly worsened by week 4. Likewise, in an open field test, the total distance traveled by the mice in the PHC group was significantly shorter than that of control mice. This is a crucial finding because this test measures an animal’s locomotor or ambulatory ability (46). In the present study, coexposure to Pb, Hg, and Cd impaired the animals’ locomotor function, whereas exposure to individual metals did not have a significant effect (except for Hg). In addition, the exploratory behavior of the mice in the PHC group markedly decreased as evidenced by the gradual decrease in the amount of time spent in the center of the open field test as the treatment duration advanced.

Similarly, in a Morris water maze test, the time spent in the platform area and the total number of platform area crossings tended to be significantly lower in all treatment groups than in controls. Although the escape latency was not significantly different between the individual metal groups and the control group, it was significantly different between the PHC group and the control group. This is consistent with previous findings that animals exposed to a mixture of Pb, Hg, and Cd took longer to find the platform, suggesting that exposure to a mixture of metals can have a detrimental effect on learning and memory (20, 25, 26). The results of these behavioral tests strongly suggest that combined exposure to these metals can cause severe neurobehavioral dysfunction, even at doses that would be ineffective or less effective with exposure to the individual metals.

Although the precise neurobiological mechanisms underlying Pb-, Hg-, and Cd-induced behavioral abnormalities are not yet fully understood, previous studies have shown that neurotransmitter systems are involved in modulating locomotion, learning, and memory processes (47, 48). There is a growing body of evidence that exposure to Pb, Hg, and Cd can disrupt the homeostasis of neurotransmitters. In particular, several reports have suggested that dopamine depletion in the striatum is a major contributing factor to motor and cognitive impairment (49, 50). In this study, the dopamine content in the striatum significantly decreased in all treatment groups, but the metal mixture exposure group exhibited a greater reduction of dopamine content than the control and single metal exposure groups. This finding may also explain the more pronounced motor and cognitive decline observed in the metal mixture exposure group than in the single metal exposure groups. Similar results have been reported in other studies, demonstrating that exposure to metal mixtures can alter dopamine levels, resulting in behavioral dysfunction (37, 51). The gene encoding TH, the rate-limiting enzyme in dopamine biosynthesis, was significantly downregulated in the PHC group compared to the control group of the present study. This finding was supported by the observation that TH protein expression was also significantly decreased in the striatum of the PHC group. In addition, TH protein levels were significantly decreased by exposure to Pb or Hg alone, but to a lesser extent than by exposure to the metal mixture. These results are consistent with the data on dopamine levels, suggesting that downregulated TH expression may be a major factor in reduction of the dopamine content.

Coexposure to Pb, Hg, and Cd elicited significant upregulation of both gene and protein expression of striatal DAT, the protein responsible for the reuptake of dopamine from the synaptic cleft (52). Previous studies have also shown that exposure to Pb or Hg increases the expression of DAT, but this upregulation is not associated with improved functionality in dopamine uptake, and metal exposure can interfere with the thiol groups of DAT disulfide bridges, leading to DAT malfunction (53). This may lead to an increase in DAT expression, either as a homeostatic mechanism to maintain stable synaptic dopamine levels or as a compensatory mechanism induced by the decline in DAT function following exposure to a metal mixture.

Previous studies have shown that changes in the serotonergic system are associated with cognitive, motor, and behavioral dysfunction (54–56). Exposure to Pb, Hg, or Cd has also been shown to affect the serotonergic system (57–60). However, no study has investigated the effects of combined exposure to Pb, Hg, and Cd on motor and cognitive function. In this study, we found that the expression of SERT, a monoamine protein involved in the reuptake of serotonin, was significantly increased following exposure to a mixture of Pb, Hg, and Cd. By contrast, exposure to any individual metal did not induce significant changes in SERT expression. These findings suggest that coexposure to Pb, Hg, and Cd may have more pronounced detrimental effects on the serotonergic system than exposure to the metals individually, resulting in impairments in cognitive, motor, and behavioral function. SERT expression is controlled by levels of intracellular calcium (61), which can be highly influenced by the presence of Pb, Hg, or Cd (30, 62, 63). Coexposure of these metals may not only alter the expression of SERT by disrupting calcium signaling but also interfere with functional groups of proteins, such as sulfhydryl and amine groups, thus affecting SERT function (30, 64) and compromising the transport of serotonin. Pb has also been shown to activate p38 mitogen-activated protein kinase, which can lead to increased expression of SERT and changes in serotonergic system function and neurobehavioral function (65).

Expression of the *TPH1* gene, which encodes the rate-limiting enzyme in serotonin synthesis, was significantly higher in the striatum of animals exposed to the metal mixture than in the control group as determined by PCR array analysis. Further supporting these data, quantitative PCR and Western blotting analysis revealed significantly higher gene and protein expression of TPH1 in the striatum of animals coexposed to Pb, Hg, and Cd than in control animals. This finding is consistent with a previous study that reported increased expression of TPH1 and TPH2 in the striatum of rats exposed to Cd, although the animals’ species, dose, and gestational age were different from those in our study (66). Although TPH1 is primarily expressed in peripheral tissues rather than in the brain, its increased expression in the striatum suggests that serotonin synthesis may be altered in this region. In addition, TPH1 is the dominant isoform in the brain during late postnatal development, and it participates in the maturation and fine-tuning of serotonergic neurons, which can influence behavioral changes (67).

The adverse effects of exposure to different metals can vary depending on the nature of the metal, the route of exposure, and the type of interaction. The total toxicity of a metal mixture can differ from that of a single metal because of synergistic, additive, or antagonistic effects (68). Interestingly, the additive effects of Pb, Hg, and Cd in this study were primarily indicated by changes in precursor enzymes (TH and TPH1) and reuptake regulation (DAT and SERT) in both the striatal dopamine and serotonin systems. In addition, although SERT expression was not significantly different between the single metal exposure groups and the control group, it was significantly different between the metal mixture exposure group and the control group as well as between each of the three single metal exposure groups. Therefore, concurrent exposure to Pb, Hg, and Cd significantly disrupts the homeostasis of the dopamine and serotonin neurotransmitter systems, which are two major systems involved in regulating motor and cognitive function. This was demonstrated by the behavioral tests, which indicated notably greater degrees of motor coordination impairment, changes in locomotor activity, and learning and memory deficits in the metal mixture group. These results indicate that the instability of the dopaminergic and serotonergic systems in the striatum is closely related to the changes in the expression of their respective precursor enzymes and transporter proteins, thereby contributing to neurobehavioral toxicity induced by mixed metal exposure. Further research is warranted to elucidate the impact of the metal mixture on striatal neurons, including aspects such as signal transduction and neuronal degeneration.

## Conclusion

This study highlights the detrimental neurotoxic effects of simultaneous exposure to different heavy metals, a prevalent scenario in cases of human exposure. Simultaneous exposure to Pb, Hg, and Cd alters striatal neurotransmitter homeostasis as evidenced by changes in dopamine content and the expression of TH, DAT, TPH1, and SERT. These changes in neurotransmitter systems can result in various types of neurobehavioral dysfunction. The present findings emphasize the need for further research of the mechanisms underlying the neurotoxicity of combinations of different metals to protect public health.

## Data availability statement

The original contributions presented in the study are included in the article/Supplementary material, further inquiries can be directed to the corresponding author.

## Ethics statement

The animal study was approved by Institutional Animal Care and Use Committee of Keimyung University. The study was conducted in accordance with the local legislation and institutional requirements.

## Author contributions

SP: Conceptualization, Formal analysis, Methodology, Writing – original draft. HK: Conceptualization, Writing – original draft, Investigation, Validation. DL: Writing – original draft, Data curation, Formal analysis, Project administration. KK: Conceptualization, Funding acquisition, Investigation, Resources, Supervision, Writing – review & editing.
