# Supplementary material for: Co-exposure to lead, mercury, and cadmium induces neurobehavioral impairments in mice by interfering with dopaminergic and serotonergic neurotransmission in the striatum
Source: Front Public Health. 2023 Nov 7;11:1265864. doi: 10.3389/fpubh.2023.1265864 (PMC10662100; doi:10.3389/fpubh.2023.1265864)
Supplement: Supplementary file 1 [file Table_1.docx]

Supplementary Material

# Supplementary Tables

**Table of Contents**

**Table S1**. Quantitative real-time PCR primer sequences

**Table S2**. PCR array results of gene expression fold regulations (fold changes) in the striatum of metal mixture-treated mice

**Table S1** Quantitative real-time PCR primer sequences (5’ to 3’)

| Gene | NCBI Gene Accession Number | Sense | Antisense |
| --- | --- | --- | --- |
| TH | NM_009377 | GCACATTTGCCCAGTTCTCC | GTACACCGGCTGGTAGGTTT |
| DAT | NM_010020 | TTGCAGCTGGCACATCTATC | ATGCTGACCACGACCACATA |
| DRD4 | NM_007878 | GTTGGACGCCTTTCTTCGTG | CCCCAGCCTTCATAAGTGGT |
| SERT | NM_010484 | GCCATCAGCCCTCTGTTTCT | TGGCTTAGAGGGGAGGAGTC |
| TPH1 | NM_009414 | TCGCTGAAACAGAAGTGTGG | CAAAGTGCCAGAAAGCACTG |
| β-Actin | NM_007393 | GCAGGAGTACGATGAGTCCG | ACGCAGCTCAGTAACAGTCC |

**Table S2** PCR array results of gene expression fold regulations (fold changes) in the striatum of metal mixture-treated mice^a^

| Refseq | Symbol^b^ | Fold Regulation | Fold Change | p-value |
| --- | --- | --- | --- | --- |
| NM_009622 | Adcy1 | 1.28 | 1.28 | 0.343 |
| NM_153534 | Adcy2 | 1.29 | 1.29 | 0.109 |
| NM_138305 | Adcy3 | 1.01 | 1.01 | 0.877 |
| NM_001012765 | Adcy5 | -1.29 | 0.78 | 0.102 |
| NM_007419 | Adrb1 | 1.13 | 1.13 | 0.252 |
| NM_007420 | Adrb2 | 1.34 | 1.34 | 0.019 |
| NM_001290818 | Grk2 | 1.10 | 1.10 | 0.443 |
| NM_001285806 | Grk3 | 1.35 | 1.35 | 0.056 |
| NM_009652 | Akt1 | 1.12 | 1.12 | 0.062 |
| NM_007434 | Akt2 | 1.00 | 1.00 | 0.980 |
| NM_011785 | Akt3 | 1.18 | 1.18 | 0.065 |
| NM_007440 | Alox12 | 1.27 | 1.27 | 0.204 |
| NM_007471 | App | 1.06 | 1.06 | 0.445 |
| NM_177231 | Arrb1 | -1.09 | 0.91 | 0.142 |
| NM_145429 | Arrb2 | 1.53 | 1.53 | 0.005 |
| NM_007540 | Bdnf | 1.64 | 1.64 | 0.090 |
| NM_007578 | Cacna1a | -1.49 | 0.67 | 0.295 |
| NM_009810 | Casp3 | 1.48 | 1.48 | 0.069 |
| NM_007668 | Cdk5 | 1.38 | 1.38 | 0.034 |
| NM_007744 | Comt | 1.07 | 1.07 | 0.227 |
| NM_133828 | Creb1 | 1.15 | 1.15 | 0.221 |
| NM_019823 | Cyp2d22 | -1.07 | 0.93 | 0.354 |
| NM_138942 | Dbh | 1.63 | 1.63 | 0.016 |
| NM_016672 | Ddc | -1.09 | 0.91 | 0.508 |
| NM_010076 | Drd1 | -1.14 | 0.88 | 0.498 |
| NM_010077 | Drd2 | -1.29 | 0.77 | 0.089 |
| NM_007877 | Drd3 | 1.22 | 1.22 | 0.334 |
| NM_007878 | **Drd4** | **1.77** | **1.77** | 0.072 |
| NM_013503 | Drd5 | -1.20 | 0.83 | 0.337 |
| NM_013642 | Dusp1 | 1.36 | 1.36 | 0.155 |
| NM_173447 | Ephb1 | -1.05 | 0.95 | 0.369 |
| NM_010234 | Fos | 1.13 | 1.13 | 0.651 |
| NM_010275 | Gdnf | -1.01 | 0.99 | 0.957 |
| NM_010277 | Gfap | -1.08 | 0.93 | 0.596 |
| NM_019497 | Grk4 | 1.20 | 1.20 | 0.129 |
| NM_018869 | Grk5 | -1.02 | 0.98 | 0.876 |
| NM_011938 | Grk6 | -1.15 | 0.87 | 0.013 |
| NM_001031667 | Gsk3a | -1.03 | 0.97 | 0.799 |
| NM_019827 | Gsk3b | 1.11 | 1.11 | 0.170 |
| NM_008308 | Htr1a | 1.07 | 1.07 | 0.399 |
| NM_010482 | Htr1b | -1.26 | 0.79 | 0.225 |
| NM_008309 | Htr1d | 1.06 | 1.06 | 0.745 |
| NM_008310 | Htr1f | 1.25 | 1.25 | 0.065 |
| NM_172812 | Htr2a | 1.29 | 1.29 | 0.013 |
| NM_008311 | Htr2b | 1.07 | 1.07 | 0.701 |
| NM_008312 | Htr2c | 1.12 | 1.12 | 0.225 |
| NM_013561 | Htr3a | 1.20 | 1.20 | 0.333 |
| NM_020274 | Htr3b | 1.75 | 1.75 | 0.004 |
| NM_008313 | Htr4 | -1.04 | 0.96 | 0.698 |
| NM_008314 | Htr5a | 1.10 | 1.10 | 0.566 |
| NM_021358 | Htr6 | -1.06 | 0.95 | 0.364 |
| NM_008315 | Htr7 | 1.02 | 1.02 | 0.701 |
| NM_010585 | Itpr1 | 1.02 | 1.02 | 0.696 |
| NM_173740 | Maoa | 1.22 | 1.22 | 0.161 |
| NM_172778 | Maob | 1.13 | 1.13 | 0.334 |
| NM_011949 | Mapk1 | 1.16 | 1.16 | 0.026 |
| NM_010444 | Nr4a1 | 1.06 | 1.06 | 0.979 |
| NM_015743 | Nr4a3 | -1.20 | 0.84 | 0.038 |
| NM_011866 | Pde10a | -1.19 | 0.84 | 0.149 |
| NM_183408 | Pde4a | 1.22 | 1.22 | 0.100 |
| NM_019840 | Pde4b | -1.18 | 0.84 | 0.050 |
| NM_201607 | Pde4c | 1.31 | 1.31 | 0.027 |
| NM_011056 | Pde4d | 1.31 | 1.31 | 0.023 |
| NM_018863 | Pdyn | -1.08 | 0.92 | 0.574 |
| NM_008839 | Pik3ca | 1.25 | 1.25 | 0.064 |
| NM_020272 | Pik3cg | -1.04 | 0.96 | 0.689 |
| NM_011110 | Pla2g5 | -1.04 | 0.96 | 0.785 |
| NM_019677 | Plcb1 | -1.09 | 0.91 | 0.426 |
| NM_177568 | Plcb2 | 1.05 | 1.05 | 0.543 |
| NM_008874 | Plcb3 | -1.04 | 0.96 | 0.669 |
| NM_144828 | Ppp1r1b | -1.10 | 0.91 | 0.550 |
| NM_008854 | Prkaca | 1.17 | 1.17 | 0.279 |
| NM_011198 | Ptgs2 | 1.31 | 1.31 | 0.265 |
| NM_153054 | Slc18a1 | 1.63 | 1.63 | 0.088 |
| NM_172523 | Slc18a2 | 1.05 | 1.05 | 0.541 |
| NM_010020 | **Slc6a3 (DAT)** | **1.76** | **1.76** | 0.012 |
| NM_010484 | **Slc6a4 (SERT)** | **2.60** | **2.60** | 0.019 |
| NM_009221 | Snca | 1.36 | 1.36 | 0.056 |
| NM_026408 | Sncaip | -1.05 | 0.95 | 0.695 |
| NM_013681 | Syn2 | 1.15 | 1.15 | 0.177 |
| NM_019911 | Tdo2 | 1.34 | 1.34 | 0.087 |
| NM_009377 | **Th** | **-2.06** | **0.49** | 0.005 |
| NM_009414 | **Tph1** | **2.06** | **2.06** | 0.098 |
| NM_173391 | Tph2 | 1.49 | 1.49 | 0.136 |
| NM_007393 | Actb | 1.00 | 1.00 | N/A |
| NM_009735 | B2m | 1.49 | 1.49 | 0.032 |
| NM_008084 | Gapdh | 1.19 | 1.19 | 0.145 |
| NM_010368 | Gusb | 1.09 | 1.09 | 0.100 |
| NM_008302 | Hsp90ab1 | 1.22 | 1.22 | 0.195 |

^a^ Metal mixture group were compared to control group (n=3). ^b^ The bold symbols represent genes exceeding the cut-off value (>1.75 or <−1.75) among 84 genes involved in dopaminergic and serotonergic neurotransmission processes.
